# Supplementary material for: UCA-YOLOv8n: a real-time and efficient fruit chunks detection algorithm for meal-assistance robot
Source: PeerJ Comput Sci. 2025 Apr 15;11:e2832. doi: 10.7717/peerj-cs.2832 (PMC12190471; doi:10.7717/peerj-cs.2832)
Supplement: Supplemental Information 1 [file peerj-cs-11-2832-s001.zip › improved_YOLOv8/docs/overrides/main.html]

{% extends "base.html" %}
{% block announce %}


{% endblock %}
