# Supplementary material for: UCA-YOLOv8n: a real-time and efficient fruit chunks detection algorithm for meal-assistance robot
Source: PeerJ Comput Sci. 2025 Apr 15;11:e2832. doi: 10.7717/peerj-cs.2832 (PMC12190471; doi:10.7717/peerj-cs.2832)
Supplement: Supplemental Information 2 [file peerj-cs-11-2832-s002.pdf]

UCA-YOLOv8n

```
------- Fei Liu -------
The version of ultralytics used in this project is 8.1.9, and __version__ is identified in ultralytics/__init__.py.
```

```
The environment of my experiment:
python: 3.8.16
torch: 1.13.1+cu117
torchvision: 0.14.1+cu117
timm: 0.9.8
mmcv: 2.1.0
mmengine: 0.9.0
```

Environment configuration

```
1. pip install ultralytics
2. Additional package installation commands required:
    pip install timm==0.9.8 thop efficientnet_pytorch==0.7.1 einops grad-cam==1.4.8 dill==0.3.6 albumentations==1.3.1 pytorch_wavelets==1.3.0 -i https://pypi.tuna.tsinghua.edu.cn/simple
    The following are mainly used dyhead must be installed packages, if the installation is not successful dyhead can not be used normally! If the execution is still unsuccessful, see the mmcv installation problem at the bottom.
    pip install -U openmim
    mim install mmengine -i https://pypi.tuna.tsinghua.edu.cn/simple
    mim install "mmcv>=2.0.0" -i https://pypi.tuna.tsinghua.edu.cn/simple

Some modules that need to be compiled to run:
    1. mamba
    2. dcnv3
    3. dcnv4
```

Comes with some file descriptions

1. train.py
   A script for training the model
2. detect.py
   Inferential script
3. improved_YOLOv8\ultralytics\cfg\datasets\fruit.yaml
   Data set path
4. improved_YOLOv8\ultralytics\cfg\models\Add
   Configuration files for different improvement models
5.

```python
import warnings
warnings.filterwarnings('ignore')
from ultralytics import YOLO

if __name__ == '__main__':
    model = YOLO('yolov8-C2f-UIB-CA-Adown.yaml')
    model.train(data=r'/root/yolov8/ultralytics/cfg/datasets/fruit.yaml',

                cache=False,
                imgsz=640,
                epochs=200,
                single_cls=False,
                batch=64,
                close_mosaic=0,
                workers=4,
                device='0',
                optimizer='SGD',

                amp=True,
                project='runs/train',
                name='exp',
                )
```

model path

data set path

Comments to the train.py file
